# Endothelial Damage and Muscle Wasting in Cardiac Surgery Patients

**DOI:** 10.7759/cureus.30534

**Published:** 2022-10-21

**Authors:** George Stavrou, Georgios Tzikos, Alexandra-Eleftheria Menni, Georgios Chatziantoniou, Aggeliki Vouchara, Barbara Fyntanidou, Vasilios Grosomanidis, Katerina Kotzampassi

**Affiliations:** 1 Leeds Institute of Emergency General Surgery, Leeds Teaching Hospitals NHS Trust, Leeds, GBR; 2 Department of Surgery, Aristotle University of Thessaloniki, AHEPA University Hospital, Thessaloniki, GRC

**Keywords:** phase angle, handgrip strength, bioimpedance analysis, skeletal muscle loss, endothelial dysfunction, cardiac surgery

## Abstract

This is a post-hoc analysis to assess the effect of anesthesia, surgical trauma, and extracorporeal circuit on endothelial integrity, microvascular permeability, and extracellular fluid balance, as well as on skeletal muscle catabolism, in patients undergoing elective cardiac surgery. We included 127 well-nourished patients undergoing “on-pump” elective cardiac surgery. One day prior to surgery (D0) and again on postoperative day 7 (POD7), body mass index, body composition assessment, hand-grip strength (HGS), and mid-upper arm muscle circumference (MAMC) were measured. Patients were assigned to early recovery (ER) and late recovery (LR) groups, depending on the duration of ICU stay (cut-off 48 hours). The magnitude of change (Δ) in all parameters studied was assessed in ER versus LR groups, regarding (i) epithelial tissue dysfunction (Δ-Extra-Cellular Water percentage (Δ-ECW%), Δ-Phase Angle (Δ-PhA)), (ii) skeletal muscle mass catabolism (Δ-Skeletal muscle mass reduction%, Δ-Hand Grip Strength (Δ-HGS) and Δ-Mid Upper-Arm Muscle Circumference (Δ-MAMC)). Baseline measurements were similar in both groups. A significant difference was observed in all Δ-parameters studied (Δ-ECW%, Δ-PhA and muscle catabolism, Δ-HGS, Δ-MAMC), the worse results being correlated to the LR group. The results raise the issue that patients with early recovery may silently have pathological conditions, continuing even on the day of discharge - further research should be planned.

## Introduction

Patients undergoing anesthesia and surgical trauma are exposed to major oxidative stress, triggering the release of a systemic inflammatory response [1-4], which is much more intense in cardiac surgery with extracorporeal circulation. This contributes to endothelial activation, disruption of endothelial integrity, and increased microvascular permeability [5], leading to leakage of plasma proteins [6] and fluid accumulation in the interstitial space [7].

Nevertheless, excessive fluid retention in cardiac surgery patients delays postoperative recovery [6], increases complication rates, and prolongs mechanical ventilation, as well as ICU and hospital stay [8], all contributing to increased mortality [9]. Thus, precise evaluation of the water balance is mandatory; both because it may affect therapeutic interventions and the planning of appropriate treatment [6,9,10].

The non-invasive bioelectrical impedance analysis (BIA) remains a reliable technique, easily available at the bedside, having the potential to independently quantify intracellular and extracellular fluid volumes and the fat-free mass (FFM) and fat mass (FM) percentages [11-13].

The well-documented occurrence of systemic inflammatory response syndrome (SIRS) after cardiac surgery, apart from its harmful action on the vascular endothelium, accelerates a profound endocrine-metabolic cascade that promotes hyper-catabolism [14-17], leading to skeletal muscle breakdown [4,18,19]. Although this hypercatabolic phase starts as early as the second postoperative day [14], in the short term, a well-nourished subject sustains minimal debilitation [19], to which, usually, no attention is paid, as long as the patient presents an uneventful postoperative course. However, this “uneventful” clinical outcome is commonly associated with measurable loss of muscle mass [4,18,19].

Fluid exsanguination and retention in the extracellular space are expressed as an increase in body weight and FFM, while at the same time, skeletal muscle wasting is expressed as a decrease in body weight and FFM. Thus, we assume that the difference observed between pre- and postoperative measurements is greater than that calculated as extracellular fluid because of the decrease in muscle weight.

In a previous study from our group, body composition analysis, by means of BIA, and handgrip strength were assessed in a cohort of 179 consecutive elective cardiac surgery patients the day before the scheduled surgery and on the seventh postoperative day; our results showed that such patients are at risk of nutritional status deterioration during their hospitalization course, which, in turn, exerts an adverse effect on the outcome [20]. Attenuation of phase angle, deterioration of fat-free mass index, and tissue edema development constitute potential surrogates for the prediction of morbidity and mortality [20].

We also found that baseline body composition parameters differed significantly between patients with normal and prolonged hospitalization in terms of FFM and FFMI. In particular, both indices were lower upon admission, in the group of patients who had a prolonged hospital stay (p < 0.05) [20].

Thus, we decided to perform a post-hoc analysis in the subgroup of well-nourished only individuals; we aimed to compare those having an uncomplicated early postoperative period and those needing prolonged mechanical ventilation and ICU stay, due to a severe complication. By using, mainly, the non-invasive technology of BIA, the pre and post-operative measurements of (a) the extracellular fluid accumulation and the phase angle (PhA), representing cellular membrane integrity, and (b) the fat-free mass, the hand grip strength, and the mid-upper-arm muscle circumference, as an index of skeletal muscle wasting, were compared.

## Materials and methods

Study population

The population for this post-hoc analysis comes from a cohort of 179 patients who were programmed to undergo elective cardiac surgery using a minimally invasive extracorporeal circulation (MiECC) pump between September 2018 and August 2019 (ClinicalTrials.gov NCT03644030). From this pool of patients, 132 individuals were selected; the unique criterion was being well-nourished according to the Malnutrition Universal Screening Tool (MUST) classification applied on initial admission. MUST is a simple screening tool for classifying subjects into low, moderate, and high risk for malnutrition based on three independent criteria: BMI score, weight loss score in three to six months, and the acute disease effect score [21]. Well-nourished patients used in the present analysis were those who had a BMI>20kg/m^2^, weight loss of less than 5% during the last five months, and zero (0) acute disease effect score, as there was no chance to be remained fasted for >5 days postoperatively. Another five were then excluded due to comorbidities, renal failure in three, and diabetes in another two; the remaining 127 patients were thus to be assessed. Diabetes and renal failure patients were excluded on the basis that they would have some degree of pre-existing microangiopathy and endothelial dysfunction, regardless of the surgical stress.

All patients were >18 years, clinically stable, not needing emergency intervention, with no congenital heart abnormality, no previous cardiac surgery, and no history of diabetes mellitus. All had serum albumin levels > 3.5 g/dL and normal renal and liver function [21]. Written informed consent was obtained from all participants.

The 127 patients were retrospectively divided into two groups based on the duration of intubation and mechanical ventilation: group ER - early recovery (extubation) - 101 patients remained intubated for < 24 hrs - practically extubated only a few hours after surgery; and group LR - late recovery (extubation) - 26 patients who remained intubated and mechanically ventilated for >24 hrs. The demographics and clinical characteristics are presented in Table 1.

**Table 1:** Demographic and Clinical Characteristics of Participants ER-GROUP: early recovery (remained intubated for < 24 hrs); LR-GROUP: late recovery (remained intubated and mechanically ventilated for >24 hrs); BMI: Body Mass Index; LVEF: Left Ventricular Ejection Fraction; MUST: Malnutrition Universal Screening Tool; EuroSCORE: European System for Cardiac Operative Risk Evaluation; CABG: Coronary Artery Bypass Graft surgery; CPB: CardioPulmonary Bypass; ICU: Intensive Care Unit

Variable	Total	ER-GROUP <24h intubation	LR-GROUP >24h intubation	p Value
Number of patients	127	101	26	
Age (y)	66.5 ± 6.2	66.2 ± 7.4	67.8 ± 4.2	0.066
Males n (%)	97 (76.3)	77 (76.2)	20 (76.9)	0.659
BMI (kg/m^2^)	22.4 ± 3.16	22.5 ± 3.21	22.1 ± 3.0	0.530
LVEF (%)	52.1 ± 8.5	52.6 ± 8.0	50.4 ± 9.9	0.159
MUST	0 (low risk – well nourished)	0	0	
EuroSCORE II (%)	2.4 (1.6-3.1)	2.3 (1.6 -3.1)	3.2 (2.3 -4.2)	0.001
Type of operation				
CABG n (%)	68 (53.54)	56 (55.44)	12 (46.15)	0.695
Valve n (%)	51 (40.15)	39 (38.61)	12 (46.15)	0.695
CABG + valve n (%)	8 (6.29)	6 (5.94)	2 (7.69)	0.695
CPB duration (min)	78.1 ± 31.4	78.4 ± 33.7	76.8 ± 29.2	0.700
Mechanical Ventilation				
<24h n	101	101	0	
>24h n	26	0	26	
ICU stay (days) median (range)	2 (1-3)	1 (1-2)	6 (3-12)	0.000
Hospital stay (days) median (range)	9 (8-11)	8 (8-9)	14.5 (12-18.2)	0.000
Mortality n (%)	3 (2.36)	0	3 (11.53)	0.010

Basic data collection

On the day of hospital admission, anthropometric (age, gender, body weight, and height) and clinical status data were registered. Body mass index (BMI) was calculated the day before surgery (D0) and patients were classified according to their MUST score [22]. EuroSCORE II was also calculated.

Body composition assessment (BIA)

Body composition was conducted by BIA on D0 and upon the seventh postoperative day (POD7). A single tetrapolar measurement was applied (QuadScan 4000, Bodystat®, Isle of White, UK) [23,24]. Then, by introducing multiple frequency currents (5, 50, 100, and 200 kHz) into the body, resistance and reactance were measured, thus enabling the estimation of FFM and FM, total body water (TBW), and intra- and extracellular fluid. These parameters were then automatically calculated by the BIA software [24] and expressed as percentages while from the raw data of resistance and reactance at 50 kHz, the phase angle (PhA) was also automatically calculated [20].

Endothelial dysfunction estimation

The magnitude of change (Δ) in extra-cellular water percentage (ECW%) was determined by subtracting the postoperative values measured on POD7 from the preoperative on D0, for both the ER and LR groups, separately, in an effort to assess the fluid, which had escaped to the extracellular space and remained there up to POD7. In the same manner, the Δ-PhA values (in degrees), obtained by subtraction of values on POD7 from that of D0, were also calculated, the reduction of absolute value generally recognized as endothelial damage [20].

Skeletal muscle wasting estimation

The muscle mass percentage at a distinct time point (D0 and POD7) was assessed by subtracting the TBW% from the FFM%, assuming that FFM% represents the sum of percentages of skeletal and visceral muscle mass, of bone mass, and of TBW%; in other words, everything but the FM%. By accepting that percentages of both bone mass and visceral muscle mass remain practically unaffected within a seven-day period, the calculated difference represents the percentage reduction of skeletal muscle mass in the body as a whole. The Δ in skeletal muscle percentage, determined by subtracting the skeletal muscle mass on POD7 from that of D0, gives the percentage of muscle loss in each group.

The hand grip strength (HGS) was measured as an indirect indicator of skeletal muscle strength and functionality, on days D0 and POD7, by using a portable hydraulic hand dynamometer (Takei 5001 GripA Analogue Handgrip Dynamometer, Takei Scientific Instruments CO, Japan); the mean value of three consecutive measurements (in Kg) from the non‐dominant hand was recorded [25,26]. The Δ-HGS obtained from the subtraction of values on POD7 from D0 in each group expresses the reduced muscle strength postoperatively.

The most commonly used method to assess muscle mass in clinical practice is the mid-upper-arm muscle circumference (MAMC) measurement (in cm). It derives from an equation correlating the mid-upper-arm circumference (MUAC), as a measure of the sum of the muscle and subcutaneous fat in the middle of the upper arm, and the triceps skin fold thickness (TSFT) as a measure of skin and subcutaneous fat thickness. Thus, MAMC = MUAC - [3,14 x TSF thickness/10], the MUAC measured in triplicate [27,28]. The TSFT measurements were performed with the Harpenden skinfold caliper (British indicators Ltd, St Albans, Herts, UK) read to the nearest 2 mm.

The Δ-MAMC obtained by subtraction of MAMC values on POD7 from D0 expresses the postoperative reduction of muscle circumference in the mid-upper arm in each group.

Clinical outcome evaluation

The length of the ICU stay and the total hospital stay, as well as the in-hospital mortality, was registered for every participant.

Statistical analysis

Descriptive data were reported as mean and standard deviation, if normally distributed, otherwise in median and interquartile (IQR, 25%-75%). The normality of data was assessed by using the Shapiro-Wilk test; in case of a violation of the normality assumption, non-parametric statistics were used.

The data were analyzed by using GraphPad Prism, 9.2.0 for Windows (GraphPad Software, San Diego, California, www.graphpad.com). For comparisons between groups, the two-tailed, unpaired t-test with Welch’s correction was used. A p-value of less than 0.05 was considered significant.

All data collected preoperatively, i.e. demographic, anthropometric, and left ventricular ejection fraction (LVEF) were divided into two groups, according to the cut-off point of 48h of ICU stay, and analyzed accordingly, to validate the matching of the patients. Similarly, TBW%, ECW%, FFM%, PhA, HGS, and MAMC preoperative values were analyzed to ensure that both groups were similar upon entry to the study. All the main parameters of the study - ECW% and PhA for endothelial damage and skeletal muscle %, HGS and MAMC for skeletal mass wasting - presented as the Δ between POD7 and D0 - were then also analyzed by the two-tailed, unpaired t-test with Welch’s correction. The chi-square test was used for the analysis of the nominal variables. A p-value of >0.05 was considered significant.

A power analysis was performed, which estimated that a sample size of at least 115 patients with an enrollment ratio of 4:1 (92 in group A and 23 in group B) would provide a power of 80% with a level of significance of 0.05 (two-sided), for detecting an increase of 25% between paired values.

## Results

Groups ER and LR consisted of 101 and 26 patients, respectively. Demographic anthropometric data and LVEF were similar in the two groups. Patients were also subjected to the same type of operation, by the same operating team, the duration of extracorporeal circulation being quite similar.

The D0 values of all parameters implicated in calculations for the final assessment of endothelial functionality and muscle mass were found to be statistically non-significant between the two groups. This finding confirms the preoperative similarity of groups, which means that whatever the difference thereafter observed, could in no way be associated with pre-existing underlying impairment of either the endothelium functionality or the FFM% (as a consequence of the muscle mass%) since the TBW% is the same (Table 2).

**Table 2:** Anthropometric Variables on Day 0 TBW% = Total Body Water %; ECW% = Extracellular Water%; FFM% = Fat Free Mass %; PhA = Phase Angle; HGS = Hand Grip Strength; MAMC = Mid-Upper Arm Muscle Circumference; ER = Early Recovery; LR = Late Recovery * Each variable is expressed as mean ± SD. ** two-tailed t-test, with Welch correction. No statistically significant difference between the early recovery (ER) and late recovery (LR) groups.

Variable*	ER GROUP <24h intubation	LR GROUP >24h intubation	p Value**
TBW%	51.4 ±6.17	53.4 ±8.04	0.22
ECW%	21.3 ±2.49	23.0 ±3.26	0.08
FFM%	67.3 ± 9.01	66.9 ±9.42	0.84
PhA⁰	5.25 ±.077	4.9 ±0.88	0.09
HGS	29.3 ±9.24	26.7 ±5.4	0.07
MAMC	30.8 ±3.19	29.7 ±2.34	0.065

Endothelial dysfunction estimation

**Figure 1:**
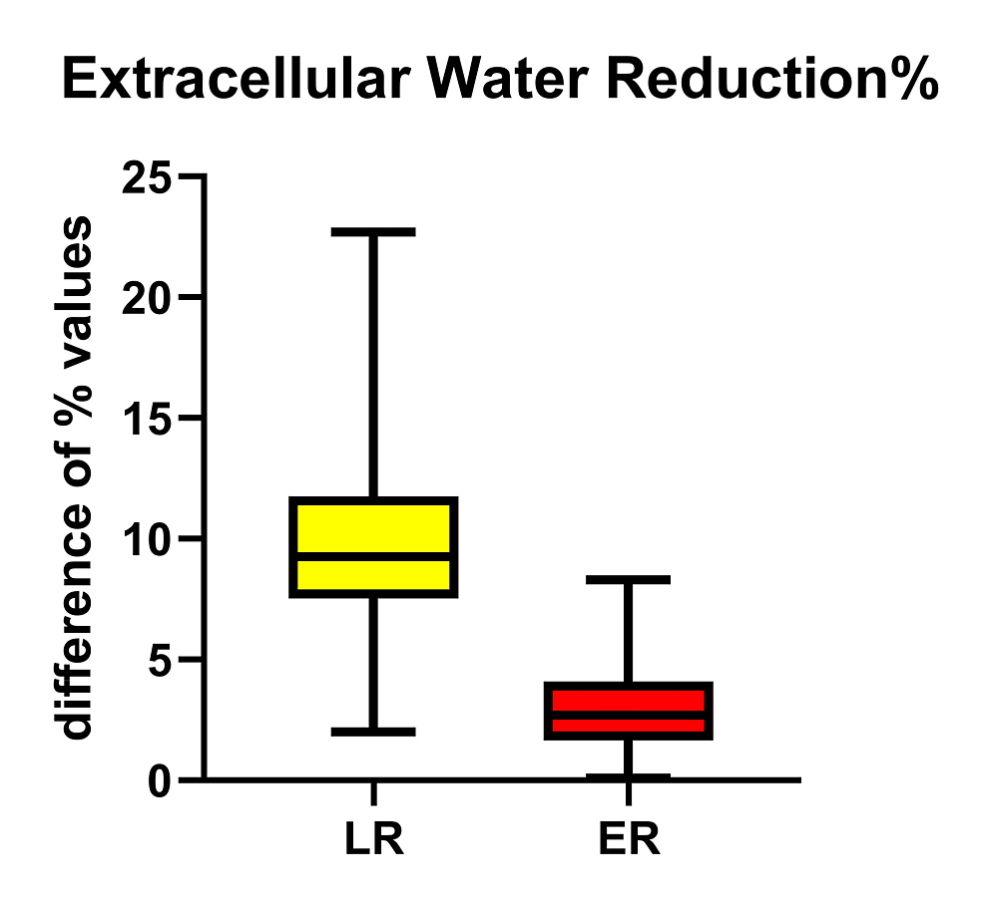
The Magnitude of Change (Δ) in Extracellular Water % (Δ-ECW%) Between POD7 and D0 in the LR and ER Groups Presented as Significantly Increased in the LR Group, Meaning Tissue Edema (p<0.0001) ER = Early Recovery; LR = Late Recovery

The Δ-ECW% between POD7 and D0 in each group reveals a statistically significant increase in the LR group in relation to ER (p<0.0001). This finding clearly demonstrates a disturbance in the endothelial function in the LR group, thus allowing the intravascular fluid to leak toward the extracellular space, and much more, to remain unmanaged up to POD7 (Table 3, Figure 1).

**Table 3:** Endothelial Dysfunction and Skeletal Muscle Wasting Δ-ECW% = Δ-Extracellular Water%; Δ-PhA = Δ-Phase Angle; Δ-SMML% = Δ-Skeletal Muscle Mass Loss%; Δ-HGS =Δ-Hand Grip Strength; Δ-MUAC = Δ-Mid-Upper Arm Circumference; ER = Early Recovery; LR = Late Recovery * Each variable value expresses the magnitude of changes (Δ) between POD7 and pre-operative measurements D0 (mean ± SD). ** two-tailed t-test, with Welch correction. A significant difference between the early recovery (ER) and late recovery (LR) groups.

Variable*	ER GROUP <24h intubation	LR GROUP >24h intubation	p Value**
Δ-ECW%	3.08 ± 1.87	9.88 ± 4.73	<0.0001
Δ-PhA	1.39 ± 0.53	1.92 ± 0.72	0.0015
Δ-SMML%	1.37 ± 1.68	5.41 ± 1.78	<0.0001
Δ-HGS	5.24 ± 3.86	10.5 ± 5.95	0.0002
Δ-MUAC	3.43 ± 1.77	11.4 ± 1.99	<0.0001

**Figure 2:**
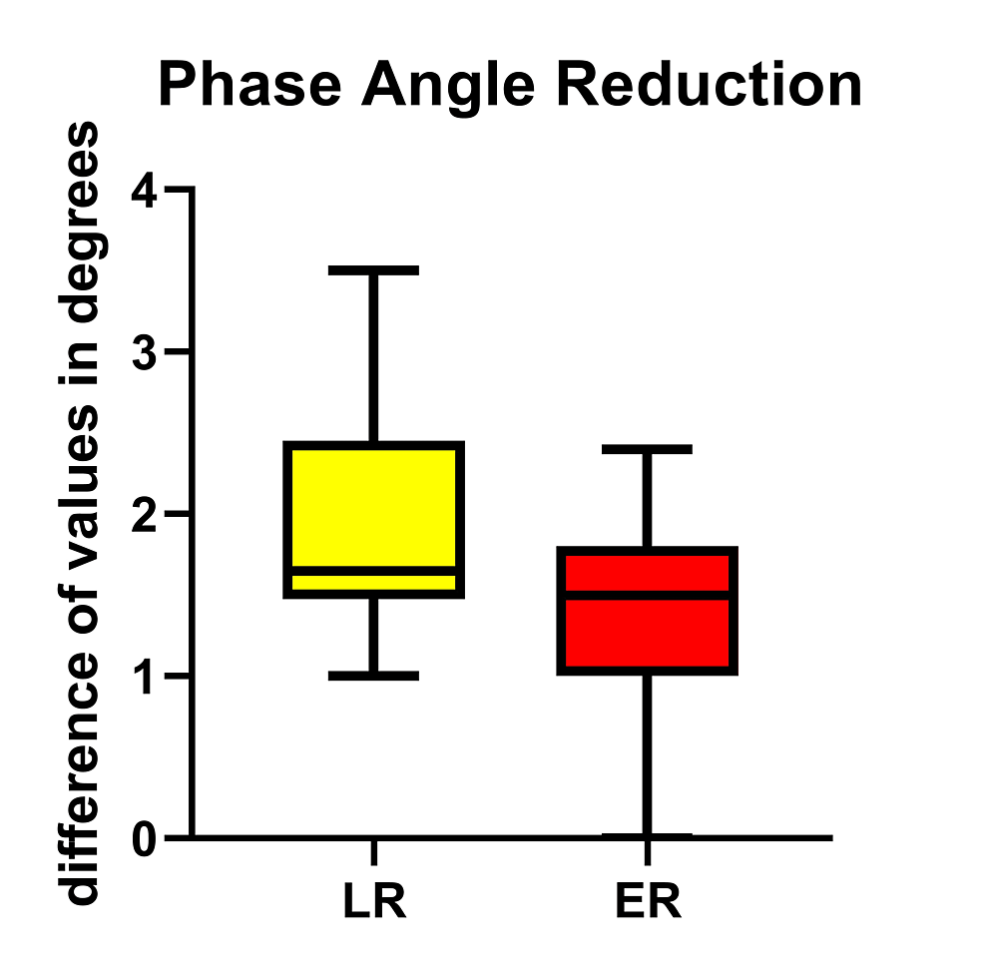
The Magnitude of Change (Δ) in Phase Angle (Δ-PhA) Between POD7 and D0 in the LR and ER Groups Presented as Significantly Increased in the LR Group, Meaning Endothelial Damage (p<0.0015) ER = Early Recovery; LR = Late Recovery

The endothelial membrane dysfunction was also directly documented by the significant increase of the Δ-PhA between POD7 and D0 in LR in relation to the ER group (p=0.0015), the reduction of the value being well-recognized as demonstrating endothelial damage and increased leakage (Table 3, Figure 2).

Skeletal muscle wasting estimation

**Figure 3:**
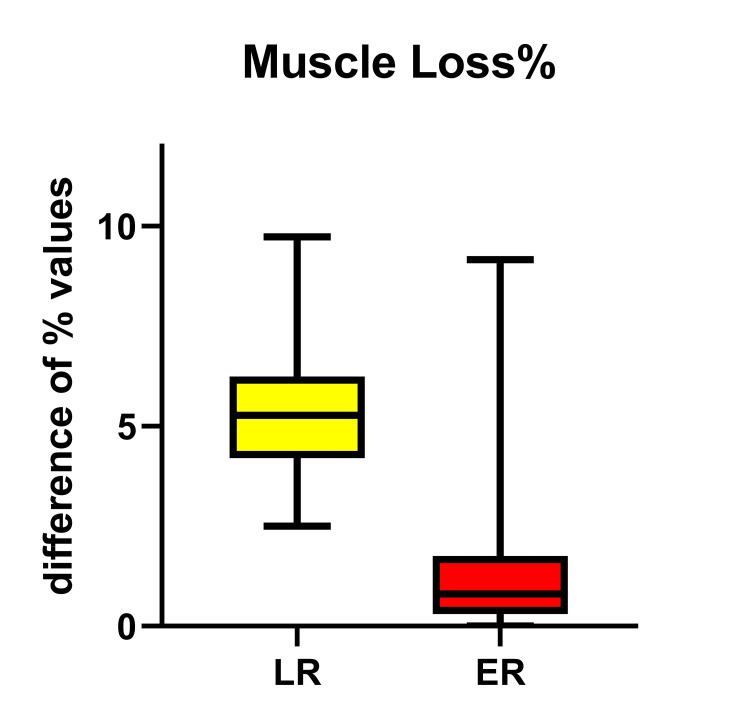
The Magnitude of Change (Δ) in Muscle Mass Loss% (Δ-SMML%) Between POD7 and D0 in the LR and ER Groups Presented as Significantly Increased in the LR Group, Meaning Skeletal Muscle Mass Wasting Postoperatively (p<0.0001) ER = Early Recovery; LR = Late Recovery

Given that FFM% and TBW% had no significant difference on D0 between groups and by accepting that the difference between them (FFM% minus TBW%) indirectly expresses percentage muscle mass alteration, our findings exhibited a significant reduction in the Δ-skeletal muscle mass loss on POD7 in the LR group in relation to ER (p<0.0001) (Table 3, Figure 3).

**Figure 4:**
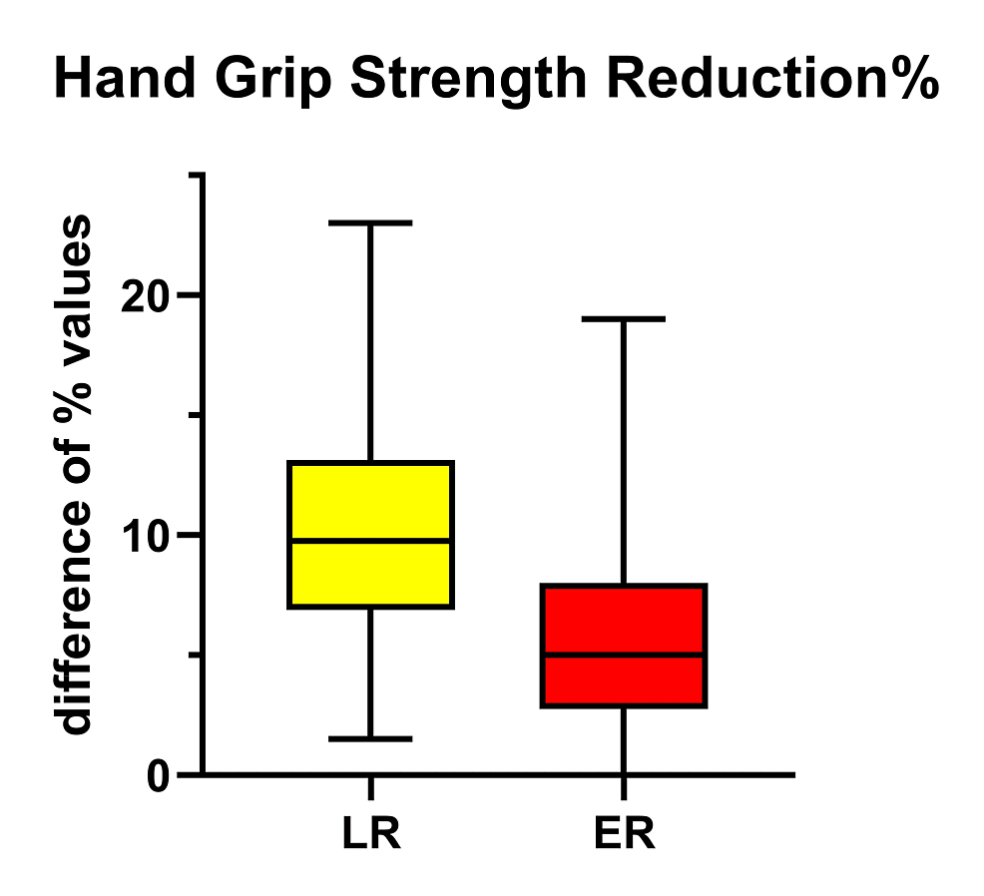
The Magnitude of Change (Δ) in Hand Grip Strength% (Δ-HGS%) Between POD7 and D0 in the LR and ER Groups Presented Significantly Increased in the LR Group, Meaning Skeletal Muscle Strength Lost Postoperatively (p<0.0003) ER = Early Recovery; LR = Late Recovery

This finding was also supported by the results of the Δ-HGS; there was a statistically significant decrease in strength, almost double in the LR group in relation to ER (p=0.002), which is also easily attributable to muscle loss% postoperatively (Table 3, Figure 4).

**Figure 5:**
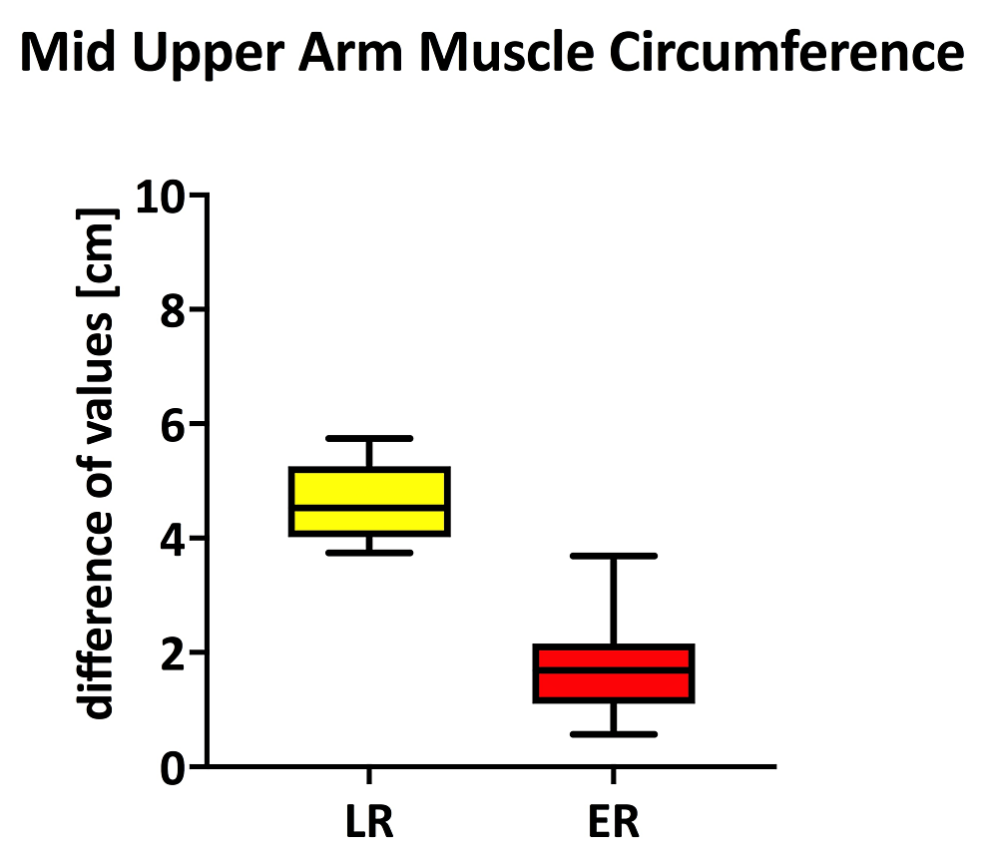
The Magnitude of Change (Δ) in Mid-Upper Arm Muscle Circumference (Δ-MAMC) Between POD7 and D0 in the LR and ER Groups Presented Significantly Increased in the LR Group, Meaning Skeletal Muscle Mass Loss Postoperatively (p<0.0001) ER = Early Recovery; LR = Late Recovery

Findings regarding Δ-MAMC were similar; there was a statistically significant decrease in MAMC between groups (p<0.0001), directly expressing a statistically significant post-operative loss of muscle mass% (Table 3, Figure 5).

Clinical outcome evaluation

The 101 patients in the ER group remained intubated for <24 hrs and in the ICU for up to 48 hrs while the 26 patients in the LR group remained in the ICU for a median of six days (range 3 to 12); the ER group spent a median of eight days in the hospital, with zero mortality, while the LR a median of 14.5 days (12 to 18.2) with three deaths (11.53%) (Table 1).

## Discussion

Clinical and experimental studies dealing with the pathophysiology of body response to tissue trauma have documented the release of SIRS [29] triggered by anesthesia and surgical trauma [1-4]. During coronary artery bypass graft (CABG) surgery with cardiopulmonary bypass (CPB) (on a heart-lung machine), this response seems to be highly augmented since, besides the anesthesia and surgical trauma-induced release of oxidative stress compounds, the harmful consequences of inevitable CPB are added even in elective operations on well-nourished individuals.

One of the most studied consequences of inflammatory response is vascular endothelial dysfunction [29,30]; the imbalance of the ANG2/ANG1 ratio toward dominance of the barrier-disruptive ANG2 in conjunction with a significant reduction in systemic NO bioavailability [5] leads to myosin-mediated contractions of endothelial cells, disruption of endothelial tight junctions, and decrease in vascular tone, and thus to net extravasation of fluid, enhancement of the transcapillary flow of plasma proteins, and collapse of microcirculation [5,29,31].

Vascular endothelial dysfunction

In the present post-hoc analysis, the first part involved the magnitude of vascular endothelial dysfunction, which remained disturbed up to POD7, by studying the ECW% and the PhA. In well-nourished patients, subjected to cardiovascular surgery, the Δ-ECW% revealed a significant increase in LR in relation to ER patients.

The decision on the selection of POD7 for the second measurement was based on the day the majority of patients were discharged from the hospital being in a generally well-managed clinical condition. This decision was also compatible with the findings of Fu et al. [12], who found that on the fifth day, their patients gained their preoperative walking abilities and their grip strength plateaued. Similarly, Gomes et al [29], stated that in their fit-for-discharge patients, post-operative endothelial dysfunction was as obscure as in other clinical situations, like diabetes, in which microvascular endothelial dysfunction is found. With respect to the cut-off point of 24hrs used for the assignment of patients to each group, we relied on the finding of the initial study, where our patients were clearly identified as those, fortunately not many, needing to remain intubated and those who extubated within a median of 24 hours [20].

Referring back to the Δ-ECW%, it should be noted that the ECW% still remained increased by almost 10% of TBW on POD7, in LR patients compared to 3% in ER. It is well accepted that in CABG patients, a large part of the procedure-related fluid shift is attributed to the use of extracorporeal circulation, although this could be partially explained by the greater frequency of urgent procedures and the higher risk of on-pump surgeries [9]. Costa et al. calculated that the ECW was significantly higher in on-pump patients than in off-pump patients [9]. However, all patients had homogeneous features: elective on-pump-only surgery and no urgent re-operation for a severe complication. Wilmore DW reported that a positive fluid balance of >3000 mL up to 24 hrs post-surgery was a reliable predictor of long-term mechanical ventilation, of >3d of ICU stay, and of longer hospital stay [19]. On the other hand, the average weight of patients was found to increase by 2 kg and the median gain for ECW was 1.1 kg [11].

Bioelectrical impedance analysis is a noninvasive technology validated at the inception of its use for the estimation of body composition, as well as the hydration status after cardiac surgery [9]. Nowadays, the availability of technologically advanced, multifrequency devices makes the direct calculation of ECW% easy, by means of the application of currents <5 kHz; this frequency proceeds predominantly through the extracellular space, whereas high-frequency signals travel through both the extra- and intracellular space, thereby enabling the difference between the multiple frequency intra- and extracellular fluids to be measured [32], thus providing a clinically useful tool for quantification of alterations in body hydration status and making it possible to predict clinical risk [10,20,33].

Another parameter measured by the new generation BIA devices is the PhA, which is used to quantify cell membrane integrity, and the extent of fluid redistribution between intra- and extracellular fluid compartments [20,32], or, in other words, whether the permeability of the cell membrane has changed [33]. A low PhA reflects a decreased cell integrity or even cell death and may be an indicator of worsening general health status and comorbidity [34]. Additionally, PhA was found to be significantly and positively related to somatic protein and muscle function [35]. Since PhA incorporates lean body mass and hydration status into a single non-invasive marker, it can serve as a surrogate to FFM for nutritional status assessment [20]; which is why Tan et al., working with hemodialysis patients, conclude that PhA is an independent predictor of protein-energy wasting, the cut-off value to predict wasting being 4.6° [36]. Although our material is totally different from that, if a hypothetical correlation could have been made, all our patients would have had a lower PhA value on POD7.

Moreover, the application of multifrequency BIA, besides the data given for the re-distribution of ECW% over a seven-day period, provided useful information in regard to endothelial cell integrity, reflecting the fluids’ trans-membrane permeability to the extracellular space. The Δ-PhA was significantly increased, that is the PhA became lower, in the LR versus the ER group, confirming endothelial dysfunction after cardiac surgery; and, for the case of POD7, the long delay in recovery may be explained by the prolonged disability of the circulation and renal circulation to manage tissue edema.

Skeletal muscle mass wasting

In the second part of the analysis, the magnitude of skeletal muscle mass wasting was investigated by using BIA technology, hand grip strength (HGS) measurement, and mid-upper-arm muscle circumference (MAMC).

Skeletal muscle, comprising 50-60% of body cell mass, represents one of the most affected organs by systemic inflammation [37], the deterioration beginning almost immediately post-surgery and increasing by postoperative catabolism [38]. However, the final concept is the active transfer of amino-nitrogen from skeletal muscle to visceral tissues, which are vital for survival. Thus, in the context of injury response, the quantity of muscle mass present in a patient at the onset of illness may determine his/her long-term ability to withstand a catabolic disease [19].

Although muscle mass can be measured by more reliable imaging techniques, such as CT, MRI, or dual-energy X-ray absorptiometry (DXA) and ultrasound, these techniques are high-cost and not readily available at the bedside. Thus, muscle mass was measured through a portable BIA device, the method having been accepted under the guidelines of the European Working Group on Sarcopenia in Older People (EWGSOP) and Asian Working Group for Sarcopenia (AWGS) [16], as giving a rapid turn-around of information at the patient's bedside.

In order to find net skeletal muscle mass in our patients, it was deemed necessary to make some assumptions and mathematical calculations, given that the BIA device used was not of the latest technology and able to assess appendicular skeletal muscle mass; thus, we used the total FFM%. By assuming that FFM% and TBW% were quite similar at D0 in both groups and on the assumption that bone% and visceral muscle mass% remain unaffected early post-operatively, we subtract TBW% from FFM%, the difference being the skeletal muscle mass%, at a given time point. This value, in percent, is completely independent of the changes in body weight and tissue edema that occur post-operatively. By using this path, we found a statistically significant reduction in the skeletal muscle mass% in the LR group, in relation to ER. This finding clearly indicates that skeletal muscle mass progressively reduces, although the rate of reduction not being linear; however, this reduction is inextricably, positively correlated with serious complications and morbidity [39], the most important being the manifestation of decreased respiratory muscle function, decreased strength and activity and prolonged convalescence [19]. However, of great interest is the reduction of skeletal muscle mass in the ER group too, confirmed also by others [18]. This skeletal muscle reduction, although little, is of great importance for the patient during the late postoperative period at home; he/she is likely to remain immobile and exhausted, which, possibly in combination with age, may finally turn into sarcopenia since low muscle mass is not routinely recognized in current practice, as the assessment of nutritional status is mainly based on overall weight loss or decreased BMI alone, which, unfortunately, does not differentiate fat mass from muscle mass.

The second parameter used for the assessment of skeletal muscle wasting is HGS. Our patients revealed a significant increase in Δ-HGS. Preoperative grip strength has been related to ejection fraction and lung function, associated with cardiovascular mortality, and cardiac surgery prognosis [12]; patients having grip recovery less than the cut-off point of 83.92% had 30-d post-discharge complications [12]; the decrease designates muscle proteolysis [38].

Currently, the “old fashioned” HGS assessment has come back to clinical practice, as it is considered a reliable and useful marker of muscle strength, apart from being low cost, portable, simple, and easy to apply. Its necessity was presented in a recent meta-analysis, where decreased HGS increased the risk of all-cause mortality by 1.88 times [40].

The third parameter used for the assessment of skeletal muscle wasting is MAMC. Our results endorse those previously obtained by means of BIA (indirectly) and HGS; there was a significant reduction in MAMC in the LR patients versus ER, although on D0, they were similar. In a recent study, the diagnostic accuracy of BIA and MAMC was assessed to detect low muscle mass, and CT was considered as the reference standard; both BIA and MAMC showed high specificity at conventional cut-offs used in screening, which makes them suitable instruments for detecting low muscle mass [27]. Similarly, MAMC has been strongly correlated with DXA-assessed lean body mass [41] as well as with the appendicular skeletal muscle mass index [42].

The strength of our analysis is that each set of measurements was performed on the same day, allowing a direct comparison of the data and that each parameter was multi-evaluated. Moreover, the magnitude of changes over time was analyzed per patient and not as the difference of the mean. However, the analysis also has some unavoidable limitations: (i) there is no biochemical or other data to rate the intensity of the operative stress triggering this outcome since the permission obtained by the institution’s ethics committee was strictly attached to a non-invasive evaluation; (ii) patients’ nutritional status was only assessed by the use of MUST classification; (iii) we do not have BIA measurements on postoperative day 1, immediately after surgery; (iv) finally a new-generation multifrequency BIA for the assessment of appendicular lean mass would be more reliable and useful, even for comparison only with MAMC.

To conclude, the results of the present analysis raise issues in two distinct directions: (i) patients who have an early recovery may silently go through pathological conditions continuing even on the day of discharge, such as disturbed endothelial function - tissue edema and skeletal muscle catabolism, which may result from a femoral fracture due to a fall up to sarcopenia; we must be alert, (ii) given that patients were mostly similar and received the same treatment, can the existence of gene differentiation, which might accelerate an augmented reaction to the same stimuli, be excluded? More extensive research should be planned.

## Conclusions

We recognize that both groups exhibited an increase in ECW% and a decrease in the PhA value, well compatible with the expected temporary endothelial damage, attributed mainly to the use of extracorporeal circulation. However, the Δ-ECW% between POD7 and D0 was found to be significantly increased in those patients remaining on ventilatory support for more days, clearly demonstrating the disturbance in endothelial dysfunction and increased leakage. In addition, a reduction in skeletal muscle mass%, which is in parallel with the reduction of MAMC and HGS was also found in both groups, as a result of the patient's exposure to overall surgical stress. Similarly, a statistically significant reduction has prominent in those remaining under ventilatory support for more days; this muscle frailty apparently affects respiratory muscle function and possibly delays extubation, which, in turn, acts destructively on muscle mass.
